# Foaming Properties of Lignosulfonates in the Flotation Process

**DOI:** 10.3390/polym15173575

**Published:** 2023-08-28

**Authors:** Jhon Chique, Lina Uribe, Marek Pawlik, Andres Ramirez, Leopoldo Gutierrez

**Affiliations:** 1 Department of Metallurgical Engineering, University of Concepcion, Concepción 4040371, Chile; jchique@udec.cl (J.C.); aramirezm@udec.cl (A.R.); 2 Water Research Center for Agriculture and Mining (CRHIAM), University of Concepcion, Concepción 4040371, Chile; luribe@utalca.cl; 3 Department of Mining Engineering, University of Talca, Talca 3480094, Chile; 4 N.B. Keevil Institute of Mining Engineering, University of British Columbia, Vancouver, BC V6T 1Z4, Canada; mpp@mining.ubc.ca

**Keywords:** lignosulfonates, foam, froth, flotation

## Abstract

The widely used technology for the selective flotation of copper and molybdenite using sodium hydrosulfide (NaSH) to depress copper sulfides creates environmental issues related to the potential emissions of toxic hydrosulfide gas (H_2_S) and bad odors. Previous studies showed that molybdenite flotation can be depressed by the action of lignosulfonates, but no significant progress has been made in studying the effect that these reagents have on the foaming/frothing phenomena in flotation. The objective of this work was to investigate the foaming properties of three samples of lignosulfonates through measurements of surface tension, foamability, bubble size distributions, and water recovery. A sugared sodium lignosulfonate (NaLS), a calcium lignosulfonate (CaLS), and a sample prepared by sulphomethylation of kraft lignin (KLS) were tested. It was found that all lignosulfonates displayed surface activity that decreased with pH and was related to the degree of anionicity and molecular weight. The NaLS lignosulfonate showed the highest dynamic foamability index (DFI) value, compared to that of the CaLS and KLS samples. The lignosulfonates tested in this study strongly affected bubble size. Water recovery tests performed using flotation experiments in a two-phase system showed that the KLS and NaLS samples had the strongest effect, which correlated with the surface tension, foamability, and bubble size results.

## 1. Introduction

Molybdenum contained in molybdenite is an important by-product of the copper mining industry. In copper mining operations, copper sulfides and molybdenite are mined together and then separated in a process which includes several stages of flotation. In the current practice, copper sulfides and molybdenite are separated by following a flotation process through which a Cu-Mo concentrate (bulk concentrate) is first generated and then treated with sodium hydrosulfide (NaSH) to depress copper sulfides. As a result, concentrates of Cu and Mo, containing 25–30% Cu and 50–52% Mo, respectively, are obtained. This process technology is widely used at an industrial level, but environmental issues related to potential emissions of toxic hydrosulfide gas (H_2_S) and bad odors are always important concerns. Thus, new, more friendly processes that incorporate non-toxic reagents are required.

Previous studies showed that molybdenite flotation can be depressed by the action of lignosulfonates (LS) [1,2,3], which are anionic polyelectrolytes with a structure in which carboxylic, sulfonic, and phenolic groups are the main molecular constituents. The results obtained by Ansari and Pawlik [1,2] indicated that naturally hydrophobic molybdenite can be strongly depressed using calcium LS of high molecular weight. Later, it was also reported that the depressing effect of LS on molybdenite increases with pH when lime is used as a pH modifier, which was explained by the interactions of these polyelectrolytes with the metallic calcium sites existing on the molybdenite particles surfaces, and that the use of potassium amyl xanthate (PAX) as a collector mitigates the depression of chalcopyrite flotation [3]. The use of LS and other polyelectrolytes as flotation reagents was also proposed in other applications, such as molybdenite and talc separation, barite depression, bitumen, and to reduce the depressing effect of clay minerals in flotation [4,5,6,7,8].

So far, the study of the use of LS to separate molybdenite and copper sulfides by flotation has been mainly focused on the interactions between these polyelectrolytes and mineral particles of molybdenite and chalcopyrite, with the main conclusion being that LS obtained by sulphomethylation of kraft lignin or kraft LS (KLS) were those that had the strongest depressing effect on molybdenite with no effect on chalcopyrite if PAX is used as copper collector [3]. However, preliminary tests using real Cu-Mo ores showed that LS also strongly modified the frothing behavior in the flotation process, and that samples of KLS had the strongest frothing effect. It must be noted that this effect may be strongly influenced by the presence of ions in solution [9], which is something that is out of the scope of this work but will be reported later. The selective flotation of copper sulfides and molybdenite using LS requires that molybdenite reports to the flotation tailings. However, molybdenite is a soft mineral that usually occurs as fine particles in mineral suspensions, thus the probability that these particles reach the concentrate by mechanical entrainment is high, which reduces selectivity. As mechanical entrainment is closely related to the amount of water that is recovered in the concentrate, which strongly depends on froth stability [10,11,12,13,14,15], it is of significance to study the effect of LS on the foaming/frothing behavior of flotation, in particular the effect of KLS. A better understanding of the effect of lignosulfonates on the frothing behavior of flotation systems will be useful in the future to adjust the air dispersion conditions of the process and the correct use and dosages of frothers [16].

The main objective of this work is to investigate the foaming properties of three samples of LS through measurements of surface tension, foamability, bubble size distributions, and water recovery in flotation, with a particular interest in understanding the effect of KLS, which proved to be the best molybdenite depressant.

## 2. Materials and Methods

### 2.1. Samples and Reagents

The study considered the use of three powder samples of LS: two of them were commercial samples of lignosulfonates obtained from Sigma Aldrich (St. Louis, MO, USA), i.e., a sugared sodium lignosulfonate (NaLS) and a calcium lignosulfonate (CaLS); the third sample, a KLS, was prepared through sulphomethylation of kraft lignin and further precipitation from black liquor of a cellulose pulp plant that processed radiata pine. The procedure for precipitation of the KLS sample is explained elsewhere [3].

Distilled water with electrical conductivity of 20 μS/cm was used in all the experiments and pH was adjusted using sodium hydroxide (NaOH) obtained from Merck (Kenilworth, NJ, USA). Methyl isobutyl carbinol (MIBC) obtained from Merck was used as a frother in some experiments.

#### Lignosulfonates Characterization

LS samples were analyzed for C, H, and N in a FISON 1108 elemental analyzer. The molar masses were measured using UV/Gel permeation chromatography equipment (Shimadzu (Kyoto, Japan)). Fourier transform infrared spectroscopy (FTIR) analyses were performed using a Nexus WQF-510A FTIR spectrophotometer by mixing the samples with powdered potassium bromide (KBr) to form pellets. The concentrations of calcium and sodium in the LS samples were measured using an Optima 5300 DV ICP-OES of Perkin Elmer (Waltham, MA, USA) [17,18,19,20]. More details on the procedures can be found elsewhere [3].

### 2.2. Surface Tension Measurements

The surface activity of the LS samples tested in this work was assessed by surface tension measurements using the Lecomte Du Noüy ring method with a Force tensiometer K6 from Krüss [15,21,22,23]. In this method, the surface tension is obtained from the determination of the mechanical force required to pull up a platinum ring of known radius (*R_R_*) from the solution surface. The relationship between the force, the ring radius, and the surface tension can be described by Equation (1) [23].

(1) $$\gamma_{LV}=\frac{Ff}{4\pi R_{R}},$$

where *γ_LV_* is the surface tension between liquid and vapor, *F* is the force necessary to detach the ring from the liquid, and *f* is the Harkins–Jordan correction factor that depends on the dimensions of the ring and on the volume of liquid displaced by pulling the ring. In all the experiments, the metal ring was kept clean by washing with distilled water and was flamed before every experiment. All the tests were performed in triplicate and the results reported in this work are average values.

### 2.3. Foamability Tests

**Figure 1:**
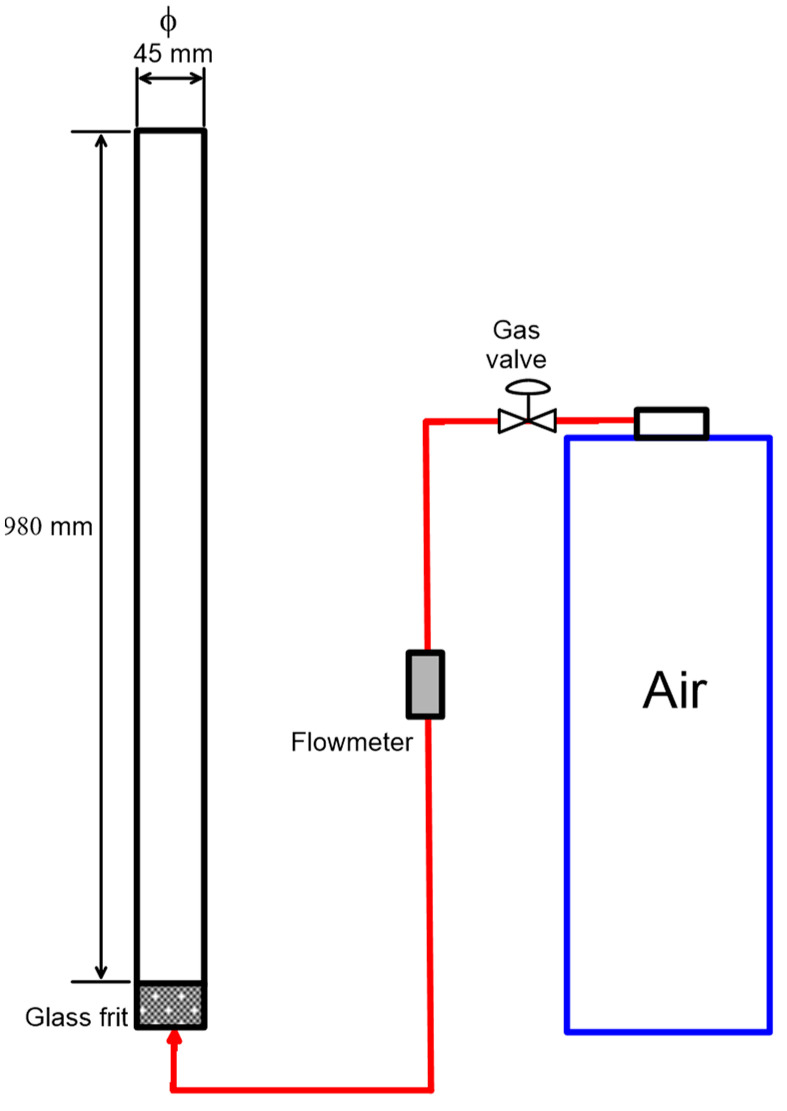
Experimental set up for foamability tests.

Foamability tests [24] were conducted using the set up described in Figure 1, which consisted of a glass column with a diameter of 45 mm (inner) and a height of 980 mm, a bubble-generator fritted glass disk, an air flowmeter, and a tank of purified air. The tests were carried out using 250 mL of LS solutions prepared at concentrations between 8 and 1600 mg/L (NaLS) and at pH values of 6 and 9. These solutions were conditioned for 15 min before being poured into the column. Then, the foam height was measured as a function of time at given values of air flowrates that varied between 0.6 and 2 L/min, which was equivalent to a range of superficial air velocity (*J_g_*) between 0.62 and 2.10 cm/s. It has to be noted that, in all the experiments, foam height reached an equilibrium at 15 min, the time at which the total gas volume in the foam was calculated as the difference between the foam volume and the 250 mL of solution. All the tests were performed in triplicate and the results reported in this work are average values.

### 2.4. Bubble Size Distributions

**Figure 2:**
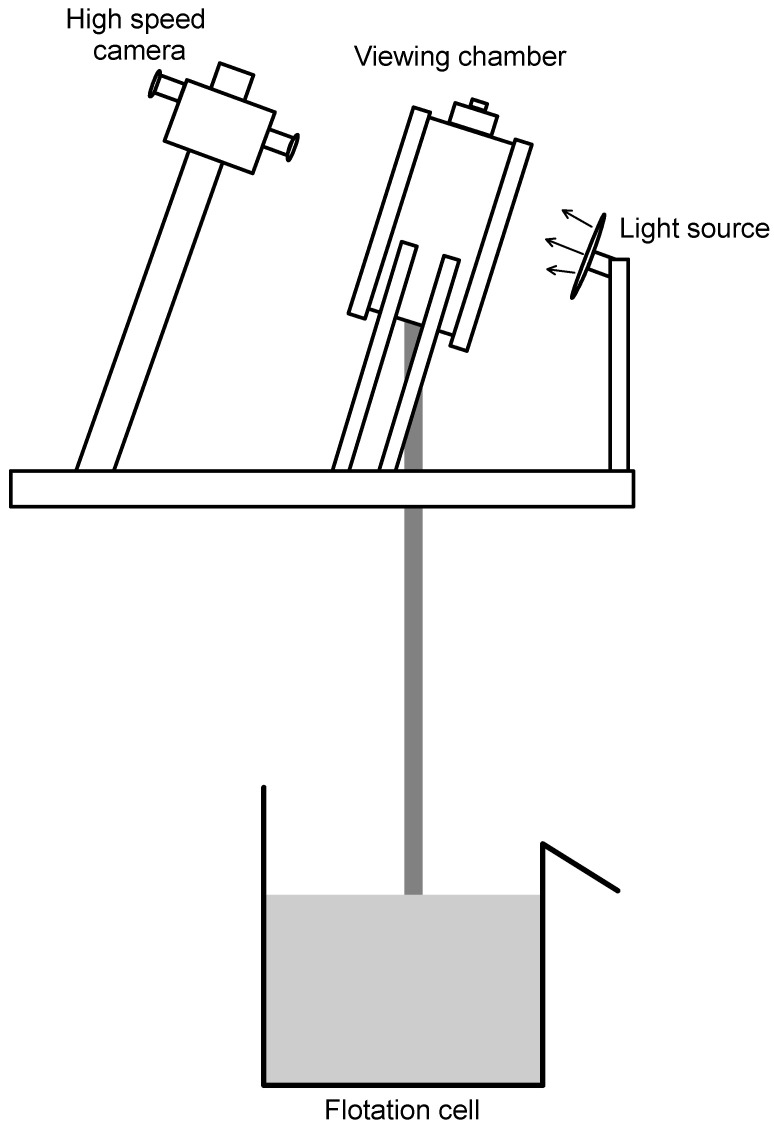
Experimental set up for measurements of bubble size distributions.

Bubble size distributions in the solution were measured using an image analysis of the bubbles, which were sampled from a flotation cell and photographed using the McGill bubble viewer [25], as described in Figure 2, which shows the experimental set up utilized for these measurements. Bubble images were captured using a Nikon D5100 digital camera (Minato ku, Japan) with a Nikon 60 mm macro lens (4928 × 3264 pixels). Bubbles were generated in a 2.7 L EDEMET flotation cell equipped with control loops to set air flowrate and agitation at 1200 rpm [26].

The bubble size analysis considered the measurement of at least 4000 bubbles to have representative samples [27]. The images obtained were processed using the Image J 1.54d software to measure the surface area of each bubble and, subsequently, the diameter (*d_i_*) of each bubble was calculated. The Sauter mean bubble diameter, *d*_32_, was then calculated according to Equation (2).

(2) $$d_{32}=\frac{\sum_{i=1}^{n}d_{i}^{3}}{\sum_{i=1}^{n}d_{i}^{2}}.$$

All the tests were performed in triplicate and the results reported in this work are average values.

### 2.5. Water Recovery Experiments

Water recovery was assessed using flotation experiments in a two-phase system consisting of LS solutions and air bubbles. Flotation tests were conducted, setting the time of foam removal to 10 s, and the foam was collected at 1, 2, 3, 4, and 5 min. Flotation tests were performed using a 2.7 L EDEMET automated flotation cell equipped with control loops to set the rate of froth removal, air flowrate, and water make-up during the tests. All the experiments were carried out at an air flowrate of 12 L/min, keeping the froth depth at 1 cm by adding solution. All the experiments were performed in triplicate and the results reported in this work are average values.

## 3. Results

### 3.1. LS Characterization

Table 1 shows the characterization of the LS samples tested in this work. The NaLS and CaLS lignosulfonates contain ~42% carbon and less than 2% of nitrogen. The sulfur contents in these commercial samples vary between 3.7 and 5.5%, and the weight-average molecular weight (*M_w_*) and number-average molecular weight (*M_n_*) range between 18,000 and 54,000 g/mol, and 2500 and 7268 g/mol respectively. The sodium content in the NaLS sample is 20.5% and the calcium concentration in the CaLS is 16.6%. The concentrations of carbon, hydrogen, nitrogen, and sulfur in the KLS are similar to those of the commercial lignosulfonates. The *M_w_* and *M_n_* of the KLS are significantly lower than for the CaLS and NaLS samples, which indicates that sulfomethylation produces a substantial fractionation of the lignin molecule.

**Figure 3:**
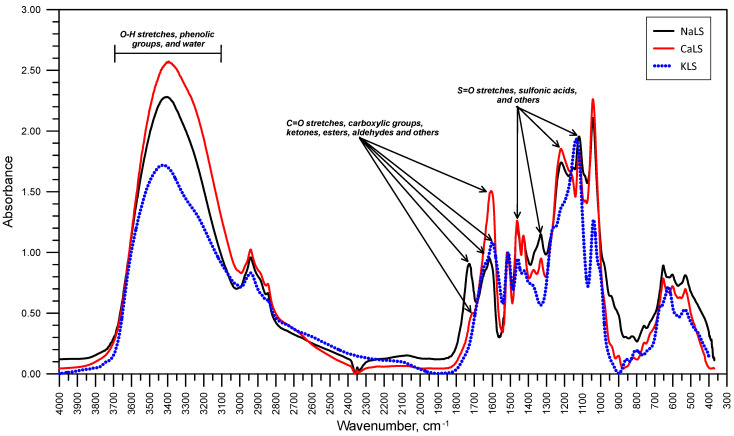
FTIR Spectra of samples NaLS, CaLS, and KLS.

Figure 3 presents the FTIR spectra of the LS samples tested in this work. There are some bands and peaks that are common for the three tested samples: the bands in the range 1600–1700 cm^−1^, which correspond to C=O stretches associated to carboxylic acids, ketones, esters, aldehydes and others; the bands at 1400–1500 cm^−1^ assigned to S=O stretches and sulfonic acids; the bands at 1100–1150 cm^−1^, which can be attributed to S=O stretching; the band at 1000–1050 cm^−1^, which relates to the presence of sulfonate groups; and the band at 3100–3700 cm^−1^, which corresponds to O-H stretches, most probably because of the presence of phenolic groups and adsorbed water or water of crystallization. It is important to note that some of the bands that appear in the FTIR spectra of the NaLS and CaLS samples are not shown in the FTIR spectra of the KLS. For example, the bands at 1700–1750 cm^−1^, which correspond to the C=O stretching vibrations of the carboxyl groups, are suppressed in the KLS sample, which also happens with the bands related to S=O stretching at 1300–1350 cm^−1^ and 1200–1250 cm^−1^ [17,18,19,20,28].

### 3.2. Surface Tension

**Figure 4:**
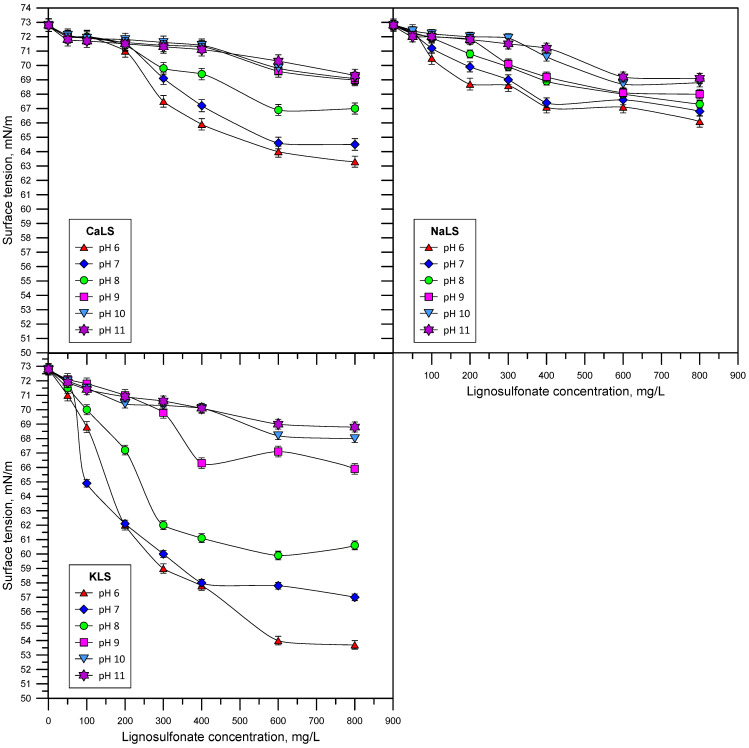
Surface tension vs. LS concentration at different values of pH for solutions prepared with CaLS, NaLS, and KLS.

Figure 4 shows the results of the surface tension measurements, obtained as a function of LS concentrations, different values of pH, and in solutions prepared with CaLS, NaLS, and KLS. The experimental results indicate that all the lignosulfonates studied in this work possess substantial surface activity in the studied concentration range, which agrees with previous studies [14,22,28,29,30,31,32,33], and that the most surface-active is the KLS, followed by CaLS and NaLS. Additionally, the surface activity of lignosulfonates is reduced as the pH of the solution increases.

### 3.3. Foamability

**Figure 5:**
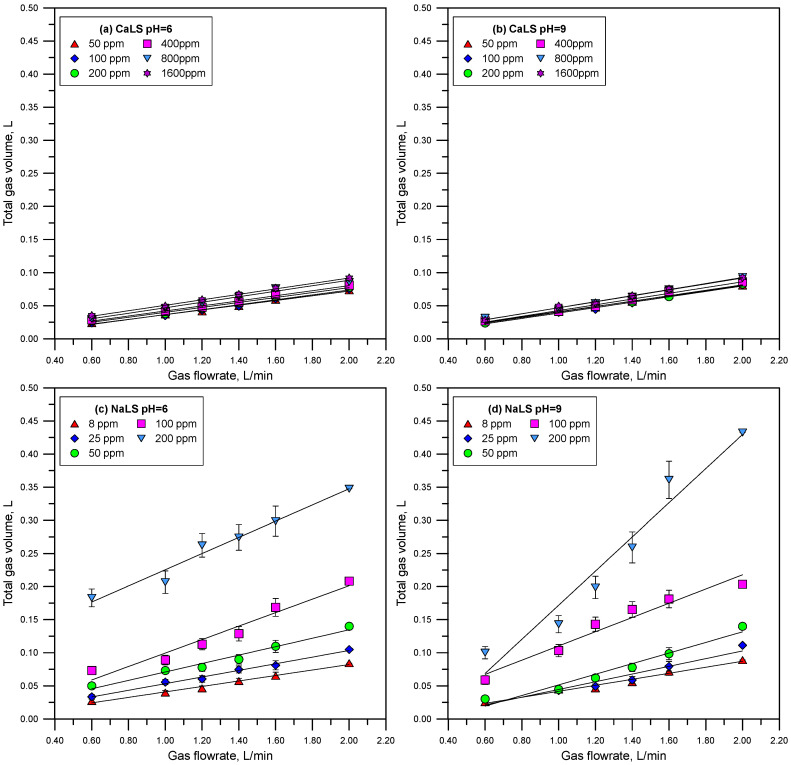
Total gas volume vs. gas flowrate at different LS concentrations (CaLS (**a**,**b**) and NaLS (**c**,**d**) samples) and at pH 6 and 9.

**Figure 6:**
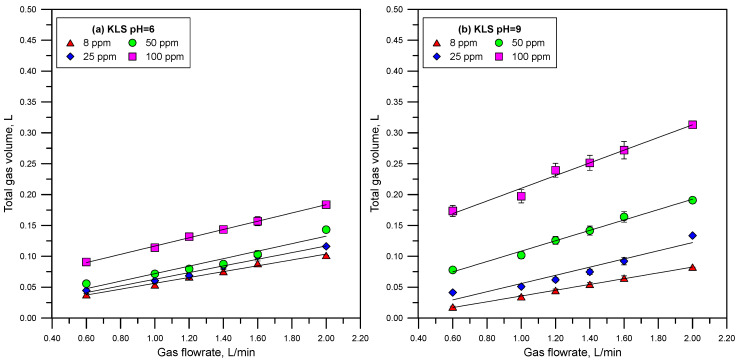
Total gas volume vs. gas flowrate at different LS concentrations (KLS sample) and at pH 6 (**a**) and 9 (**b**).

**Figure 7:**
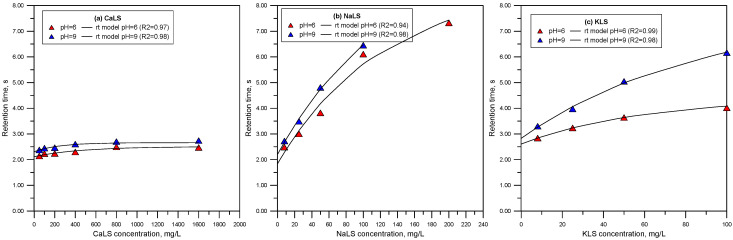
Retention times vs. LS concentration (CaLS (**a**), NaLS (**b**), and KLS (**c**) samples) at pH 6 and 9.

Figure 5 and Figure 6 show the total gas volume vs. gas flowrate curves measured using solutions of CaLS, NaLS, and KLS. It is worth noting that the relationship between gas flowrate and volume deviates from linearity only in the tests involving the NaLS sample. This deviation could be attributed to fluctuations resulting from the experimental work, which correlates with the highest experimental errors observed with this lignosulfonate sample. Figure 7 shows the retention times obtained from the straight-lines slopes of Figure 5 and Figure 6. The dynamic foamability index (DFI) can be used to classify the foaming effect of LS reagents [24,34]. DFI can be obtained from the slope of the gas retention time (rt) versus LS concentration as this concentration tends to zero. Czarnecki et al. [34] proposed Equation (3) to improve the determination of DFI from the experimental data.

(3) $$rt={rt}_{0}+{rt}_{\infty }\left(1-e^{-kc}\right),$$

where *rt*_0_, *rt_∞_*, and *k* are empirical constants and *c* is the reagent concentration. It must be noted that *rt_∞_* is the value at the limiting condition of c → *∞.* The DFI values can be calculated as the product between *rt*_∞_ and the constant *k* [34]. Table 2 shows the DFI and *rt_∞_* values obtained from the experimental data. The results show that the NaLS reagent is the one with the highest DFI value, which is higher than the results obtained using the CaLS and KLS samples and higher than reported values for MIBC of around 37,000 sL/mol [33,35]. On the other hand, the results obtained for *rt_∞_* show that NaLS reaches the highest values. It is interesting to note that both parameters, DFI and *rt_∞_*, increase with pH.

### 3.4. Bubble Size

**Figure 8:**
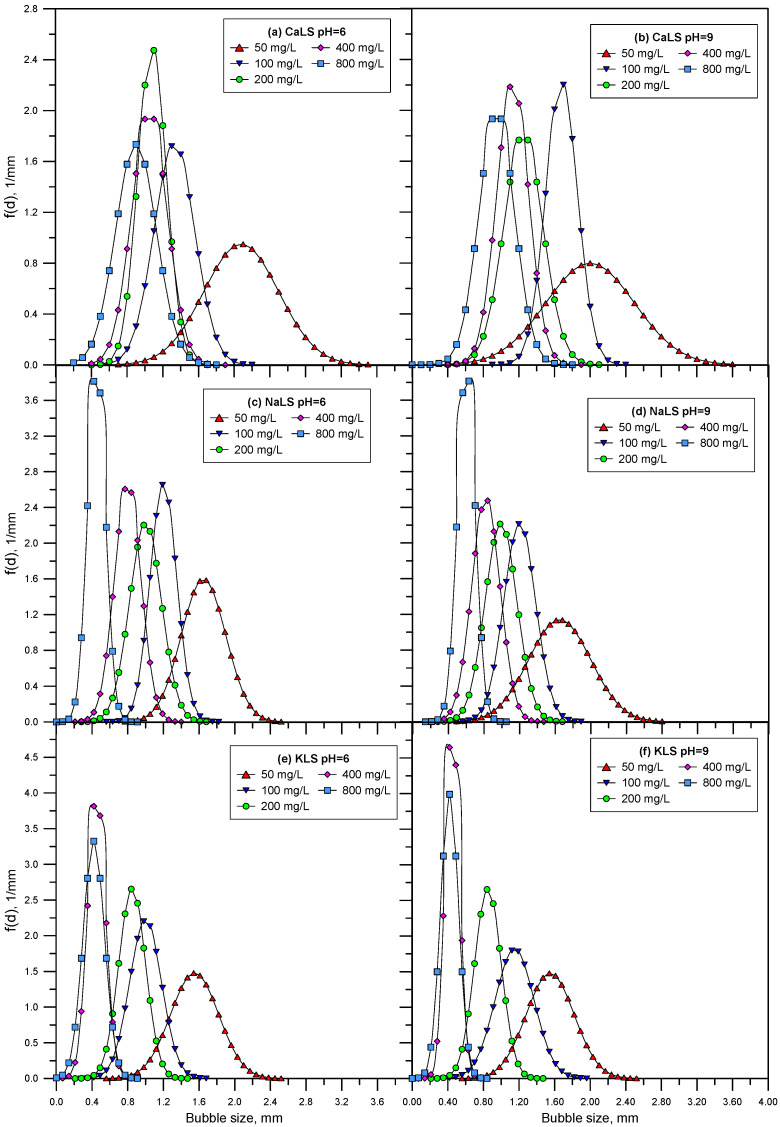
Bubble size distributions obtained using different LS (CaLS (**a**,**b**), NaLS (**c**,**d**), and KLS (**e**,**f**) samples) at pH 6 and 9.

**Figure 9:**
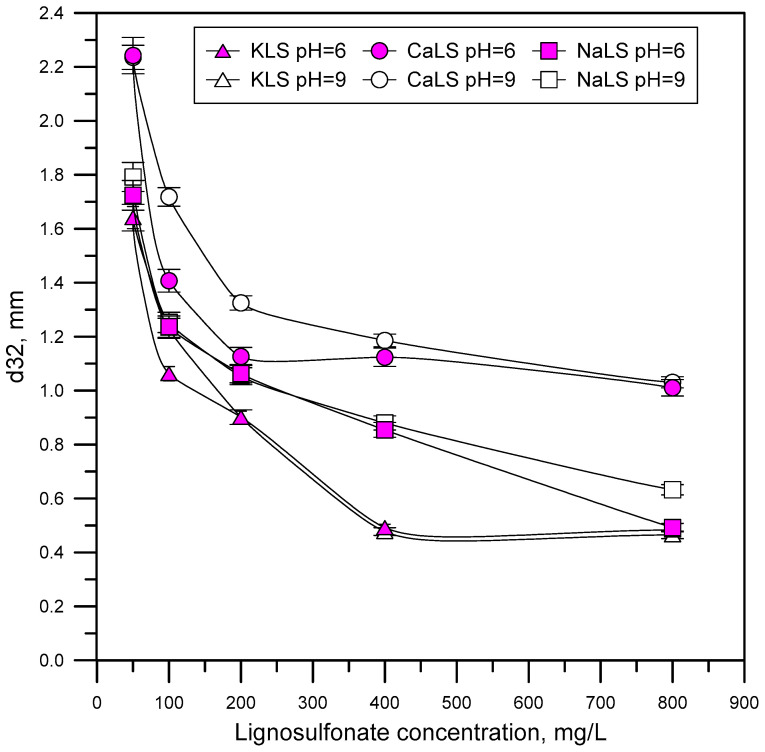
Average bubble sizes obtained using different LS (CaLS, NaLS, and KLS samples) at pH 6 and 9.

**Figure 10:**
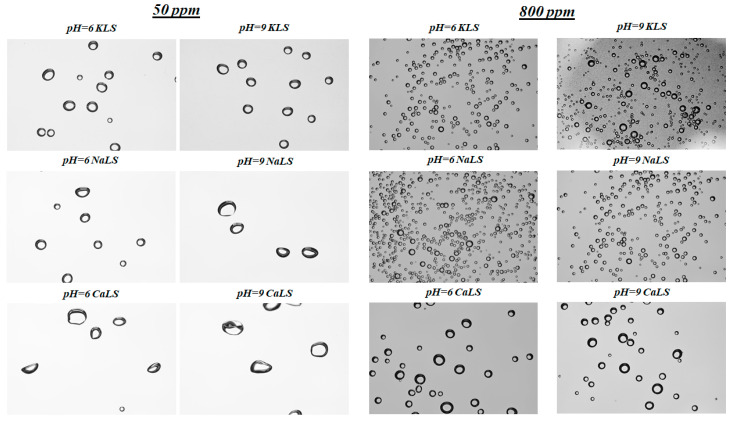
Images of bubbles obtained using different LS (CaLS, NaLS, and KLS samples) at pH 6 and 9.

Figure 8 shows the bubble size distributions obtained using different LS samples (CaLS, NaLS, and KLS samples) at pH 6 and 9. Figure 9 shows the Sauter mean bubble diameter D32 at pH 6 and 9 as a function of LS concentration, and Figure 10 shows some images of the bubbles obtained in the experiments. The results show that the KLS is the one that generates the smallest bubble sizes (minimum 0.5 mm), followed by NaLS (minimum 0.5 mm), while CaLS is the one that generates the coarsest bubbles (minimum 1.5 mm). It is important to note that the bubble size values obtained in this study using LS are like those previously obtained using MIBC at 50 ppm in the same type of cell and under similar stirring conditions [26].

### 3.5. Water Recovery

**Figure 11:**
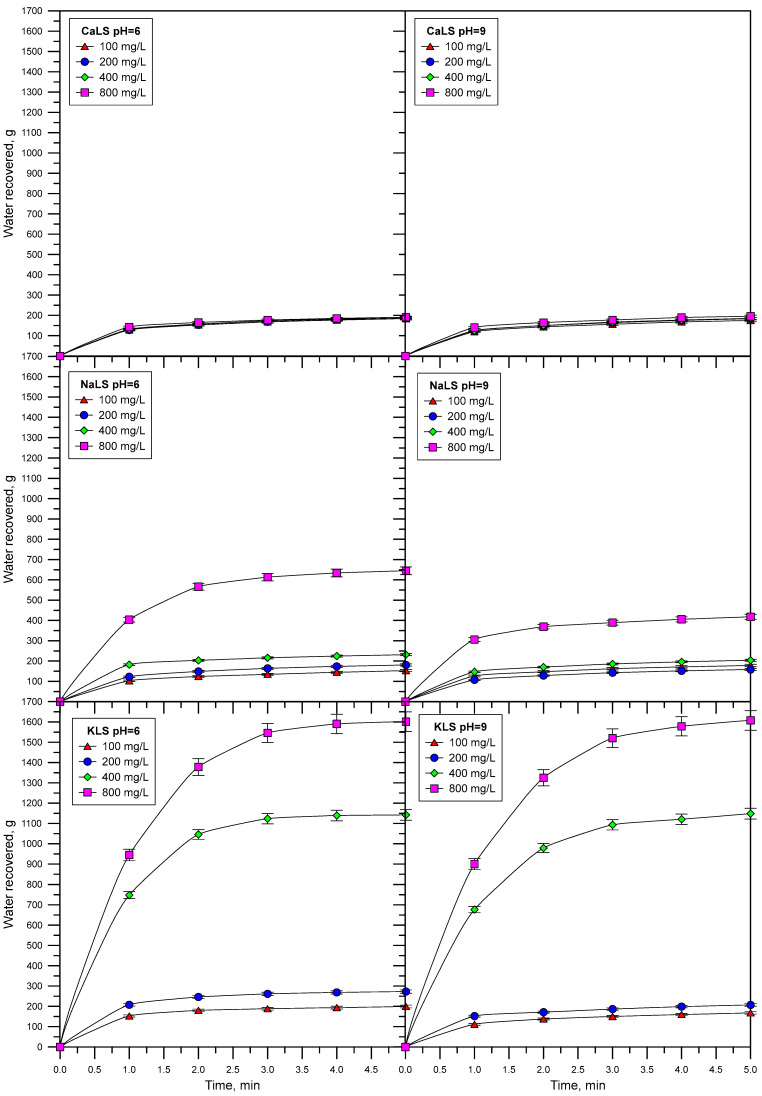
Water recovered as a function of time using different LS (CaLS, NaLS, and KLS samples), and at pH 6 and 9.

**Figure 12:**
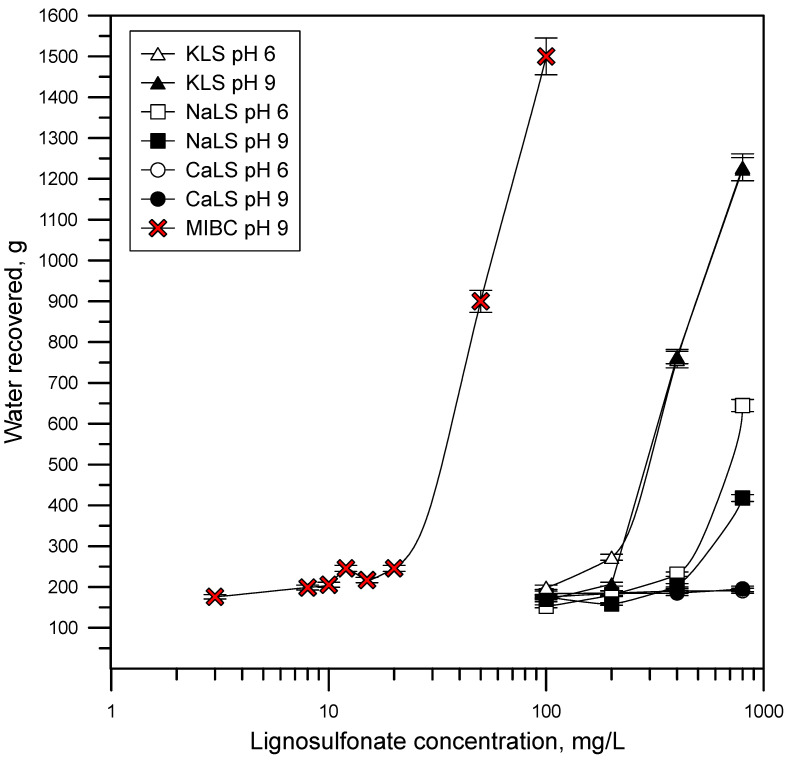
Water recovered at 5 min using different LS (CaLS, NaLS, and KLS samples), and at pH 6 and 9.

Figure 11 shows the results of the water recovered as a function of time using different LS samples, and at pH 6 and 9. Figure 12 shows the amount of water recovered at 5 min of flotation at different LS concentrations. The results show that water recovery strongly increases using the KLS and NaLS samples but not in the presence of CaLS.

## 4. Discussion

The anionic character of lignosulfonates is closely related to the structural conformation of these polyelectrolytes and depends on the degree of dissociation of the carboxylic, sulfonic, and phenolic groups. As a result of the dissociation of these functional groups, lignosulfonate molecules become more negatively charged at high pH [36]. It is well known that, for a molecule to have surface active properties, it needs to have lyophobic (no affinity with water) and lyophilic (affinity with water) molecular components, and thus the surface activity of the molecule depends on the relative concentrations of both molecular groups [37]. The experimental results presented in Figure 4 show that the surface tension decreases with the lignosulfonate concentration for the three tested samples, and that this effect decreases as pH increases. As previously indicated, lignosulfonate molecules become more negatively charged as the pH increases, and therefore the interactions with water molecules are intensified because of the increase in hydrogen and dipole–dipole bonding that rise under these conditions. Then, it is reasonable to expect that the adsorption of lignosulfonate molecules at the air/liquid interface decreases at higher values of pH. There are also studies that indicate that gas bubbles themselves have more negative zeta potentials at a higher pH [38], so an electrostatic repulsion effect between anionic LS and the negatively charged air/liquid interface could take place, leading to lower adsorption at a high pH. Figure 4 also shows the most surface active is the KLS, followed by the CaLS and NaLS. The degree of anionicity (DA) of the lignosulfonates tested in this work is proportional to the sum of the moles of positive charges related to the presence of sodium and calcium in the polyelectrolytes, as presented in Equation (4).

(4) $$DA\propto \sum_{i}Z_{i}C_{i}$$

where *Z_i_* is the cation valence and *C_i_* is the molar concentration of the cation. Considering the data presented in Table 1, the values of the term on the right side of Equation (4) for the lignosulfonates KLS, CaLS, and NaLS are 0.5, 1.05, and 0.96, respectively. According to these results, KLS is the lignosulfonate with the lowest DA and therefore the one that interacts the least with water molecules, which explains its high surface activity. At the same time, KLS is the sample that has the lowest molecular weight, which enhances molecular diffusion of these molecules towards the air/liquid interface. Lignosulfonates CaLS and NaLS have the highest DA, but since NaLS has a much higher molecular weight than CaLS, it is expected that the migration of NaLS molecules towards the air/liquid interface and surface activity should be lower. It is also important to note that divalent cations, such as calcium, can influence changes in the surface activity of CaLS. Figure 4 illustrates that the surface tension pattern of CaLS remains unchanged from pH 9 to pH 11, a range in which the hydroxo complexes of this divalent cation begin to increase. These results suggest that the nature of the counterion might impact lignosulfonate association, resulting in alterations in surface activity. This aspect falls beyond the scope of this paper and will be addressed in future research. It is also crucial to highlight that the KLS sample was used without undergoing cleaning after its extraction from kraft lignin. Consequently, there may be residual sugars in the sample that could also be impacting its surface activity. This topic will be studied in further research.

**Figure 13:**
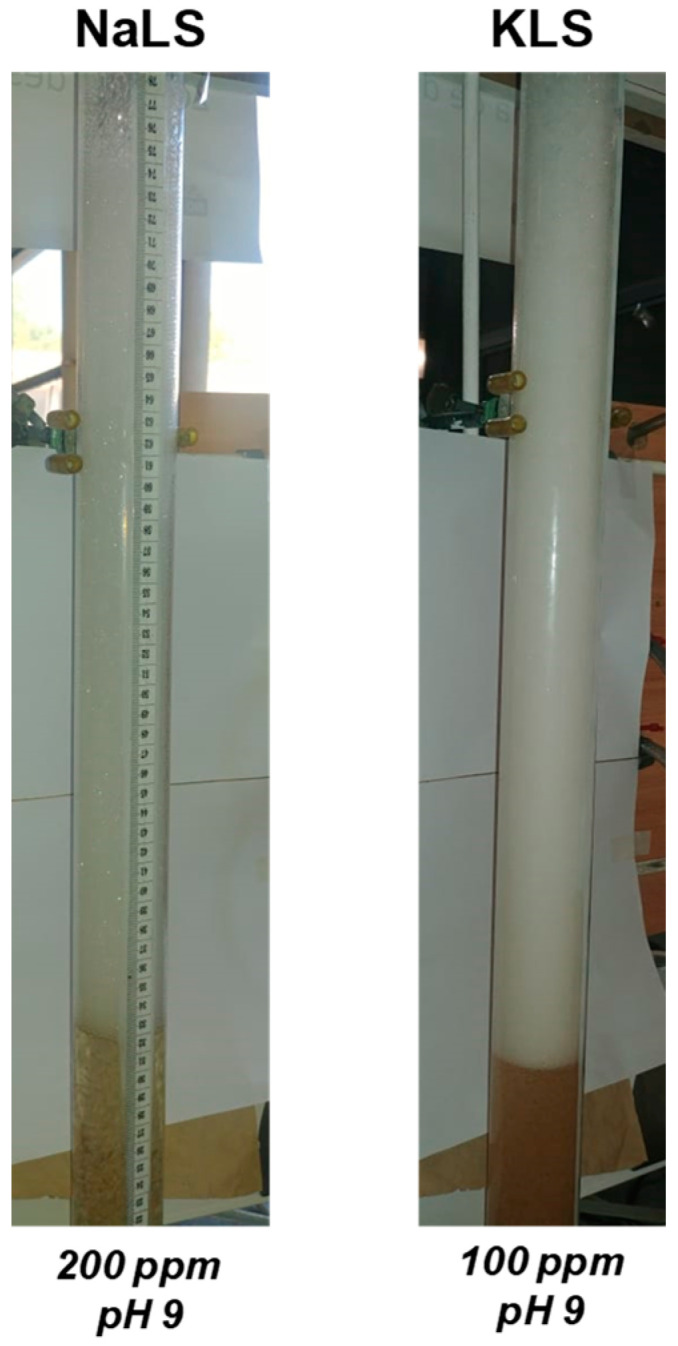
Foams obtained using different lignosulfonates (NaLS and KLS samples).

The results presented in Figure 5, Figure 6 and Figure 7 show that the NaLS and KLS lignosulfonates have the strongest foamability effect, while CaLS does not have a significant effect on foam formation. The results of bubble sizes (Figure 8 and Figure 9) show that the KLS lignosulfonate strongly inhibits bubbles coalescence, followed by the NaLS and CaLS lignosulfonates. It was well established that foams are formed by three zones, i.e., a bubble zone, a kugelschaum or sphere zone, and a polyederschaum or polyhedral zone [39]. Figure 13 shows an example of the foam obtained in the experiments with the LS samples tested in this work. In the bubble zone, bubbles move freely upwards and the gas concentration is relatively low; then, in the sphere zone, bubbles still display nearly spherical geometry and are separated by thick films of liquid; in the polyhedral zone, bubbles are separated by thin flat liquid films or lamellas, and the junction points of the interconnecting channels existing between the bubbles are known as Plateau borders [39].

The foaming phenomena occur because of the non-uniform concentration distribution of surface-active agents at the air/liquid interface, which induce resorting forces that tend to re-establish the thickness of the lamellas [39], which is closely related to bubble coalescence in the foam and the bursting of bubbles as they approach the top section of the column [8,9,10,11]. These resorting forces are referred to as the Gibbs–Marangoni effects, which are related to a non-equilibrium distribution of surfactant in the lamellas. The drainage process from the polyhedral to the bubble zone strongly depends on gravity effects and on the phenomena occurring in the lamellas and plateau borders. Due to the curvature of the air/liquid interface in the plateau borders, the pressure in the liquid phase is lower than the pressure in the lamellas, thus liquid mass transfer takes place towards the plateau borders [40]. The drainage rate also depends on other variables such as liquid viscosity and electrostatic repulsive forces existing between the air/liquid interfaces, among others. For small film thicknesses, electrostatic repulsive forces stabilize the lamella and, as a result, improve foam stability. The liquid drainage displaces surfactant molecules from the lamellas to the plateau borders, which induces a surface tension gradient and the surface tension in the lamellas decreases with respect to the surface tension in the plateau borders. Thus, liquid mass transfer from the plateau borders to the lamellas takes place because of the Gibbs–Marangoni effects, which increases the thickness of the lamellas and therefore improves foam stability. The strong foamability generated by the NaLS and KLS tested in this work can be correlated to the small bubble sizes observed, which relate to a decrease in bubble coalescence due to steric interactions between bubbles caused by the adsorption of LS molecules at the air/liquid interface; based on the results of surface tensions obtained in this work, it is plausible to think that foam stability can also be related to the manifestation of the Gibbs–Marangoni effects. The experimental results also indicated that foams generated by NaLS and KLS samples lasted for even more than 10 min after stopping aeration, which indicates a strong reduction of bubbles bursting on the top section of the foam and strong air/water interface stabilization. This topic certainly deserves further study.

The water recovery data obtained in this work are closely related to the previously discussed results. Figure 11 and Figure 12 show that the amount of water recovered strongly increases in the presence of KLS and NaLS, which correlates with the surface tension, foamability, and bubble size results. The recovery of liquid from flowing foams was studied by Neethling et al. [12], who developed and validated a model based on the physics of foams to describe the liquid recovery. The model indicates that the amount of water recovered from a flowing foam decreases as bubble size increases in the foam because of more bubble coalescence, which is inversely proportional to the square of the plateau border’s length. The same group of researchers showed later that the amount of water that overflows the foam strongly depends on foam stability, which is closely related to coalescence and the bursting of bubbles as they get to the lip column [10,11,12,13]. The authors quantified the bubbles bursting as the fraction of air entering the foam that finally overflows the column as unburst bubbles, which was referred to as α or air recovery [11]. According to these theories, the increase of water recovery obtained in the presence of KLS and NaLS is explained by the inhibiting effect of these polyelectrolytes on bubble coalescence and most probably on bubble bursting.

## 5. Conclusions

1. The lignosulfonates studied in this work displayed substantial surface activity. The most surface-active lignosulfonate was KLS, followed by CaLS and NaLS. The order of surface activity of the lignosulfonates tested was related to the degree of anionicity and molecular weight of these polyelectrolytes.
2. The surface activity of lignosulfonates decreased with the increase of pH, which is explained by the increase of negatively charged lignosulfonates at a higher pH, and by the stronger electrostatic repulsion between the more anionic lignosulfonates and the negatively charged air/water interface.
3. The NaLS lignosulfonate is the one with the highest DFI value, which is higher than the DFI values obtained for the CaLS and KLS samples, and higher than the values reported for common industrial frothers.
4. The lignosulfonates tested in this study had a strong effect on bubble size, with values like those previously obtained using MIBC. The KLS lignosulfonate generated the smallest bubble sizes (D32~0.5 mm), followed by NaLS (D32~0.5–0.6 mm) and CaLS (D32~1.0–1.1 mm).
5. The experiments performed to assess the effect of lignosulfonates on water recovery, which were carried out through flotation experiments in a two-phase system, showed that the KLS and NaLS samples had the strongest effect. The amount of water recovered strongly increased in the presence of KLS and NaLS, which correlated with the surface tension, foamability, and bubble size results. According to these results, the increase of water recovery obtained in the presence of KLS and NaLS is explained by the inhibiting effect of these polyelectrolytes on bubble coalescence and most probably on bubble bursting.

## Figures and Tables

**Table 1:** Characterization of LS samples NaLS, CaLS, and KLS.

	Moisture	C	H	N	S	Ca	Na	Sulfonate Sulfur	Carboxylic Groups	M_wa_	M_n_	M_w_/M_n_
	%	%	%	%	%	%	%	%	%	Da	Da	
NaLS	9.5	42.2	4.75	˂2.2	3.73	1.48	20.48	3.5	4.5	54,000	7300	7.40
CaLS	10.2	41.8	5.30	˂2.2	5.50	16.60	5.00	4.7	6.4	18,000	2500	7.20
KLS	9.4	38.8	4.25	3.90	5.70	0.00	11.55	5.6	6.3	960	634	1.51

**Table 2:** DFI values obtained with NaLS, CaLS, and KLS.

	DFI, sL/mol		*rt_∞_*, s		*k,* L/mol	
	pH = 6	pH = 9	pH = 6	pH = 9	pH = 6	pH = 9
NaLS	3,074,122	3,298,648	8.8	10.2	349,332	323,397
CaLS	15,304	26,310	2.5	2.7	6122	974
KLS	30,802	56,911	4.4	7.7	7000	7391
